# Energetic and Molecular Water Permeation Mechanisms of the Human Red Blood Cell Urea Transporter B

**DOI:** 10.1371/journal.pone.0082338

**Published:** 2013-12-20

**Authors:** Slim Azouzi, Marc Gueroult, Pierre Ripoche, Sandrine Genetet, Yves Colin Aronovicz, Caroline Le Van Kim, Catherine Etchebest, Isabelle Mouro-Chanteloup

**Affiliations:** 1 Institut National de la Transfusion Sanguine, Paris, France; 2 Inserm, UMR_S665, Paris, France; 3 Université Paris Diderot, Sorbonne Paris Cité, Paris, France; 4 Laboratory of Excellence GR-Ex., Paris, France; University of Pittsburgh, School of Medicine, United States of America

## Abstract

Urea transporter B (UT-B) is a passive membrane channel that facilitates highly efficient permeation of urea. In red blood cells (RBC), while the major function of UT-B is to transport urea, it is assumed that this protein is able to conduct water. Here, we have revisited this last issue by studying RBCs and ghosts from human variants with defects of aquaporin 1 (AQP1) or UT-B. We found that UT-B's osmotic water unit permeability (pf_unit_) is similar to that of AQP1. The determination of diffusional permeability coefficient (P_d_) allowed the calculation of the P_f_/P_d_ ratio, which is consistent with a single-file water transport. Molecular dynamic simulations of water conduction through human UT-B confirmed the experimental finding. From these results, we propose an atomistic description of water–protein interactions involved in this permeation. Inside the UT-B pore, five water molecules were found to form a single-file and move rapidly along a channel by hydrogen bond exchange involving two critical threonines. We further show that the energy barrier for water located in the central region coincides with a water dipole reorientation, which can be related to the proton exclusion observed experimentally. In conclusion, our results indicate that UT-B should be considered as a new member of the water channel family.

## Introduction

Aquaporin 1 (AQP1) and urea transporter (UT-B) are integral membrane proteins, both expressed in erythrocytes, allowing a rapid transport of water and urea across red blood cell (RBC) membranes, respectively [1], [2]. These functions protect RBCs from osmotic stress when they pass through the vasa recta in the medulla where urea accumulates in high concentration [3]. In addition, AQP1 and UT-B expressed in kidney epithelial cells play an important role in the control of osmotic gradient necessary for maximal re-absorption of water [2], [4], [5]. Humans lacking UT-B or AQP1 exhibit a reduced capacity for urine concentration [6]. The analysis of water transport in AQP1-deficient RBCs suggested the presence of a second pathway for water involving an unidentified protein [2]. A previous work concluded to a water transport by UT-B injected oocytes, although not under the physiological expression level of the channel [7]. By generating double knockout mice lacking both UT-B and AQP1, Yang and Verkman proposed the presence of an aqueous pore through UT-B involved in water transport [8].

AQP1 was the first water channel to be functionally and structurally characterized [9], [10]. This protein carrying the Colton (Co) blood group antigen is a tetramer. Each monomer allows a high flux of water (10^9^–10^10^ molecules per second), and, by excluding protons, preserves the electrochemical potential across the RBC membrane [11], [12]. Among several characteristic structural features of AQPs [12]–[14], there are two highly conserved regions called NPA motifs with three amino acid residues (Asn-Pro-Ala). Molecular dynamic (MD) simulations have provided an atomistic description of the mechanism of water permeation and helped in interpreting proton exclusion through AQP1 [15], [16]. Regarding the proton exclusion process, two mechanisms are put forward. The first one is related to the so-called Grotthuss mechanism, [17], in which the proton hops along a water network. This network consists of a continuous chain of hydrogen bonded water molecules. The MD pioneer work of de Groot & Grubmuller [18] showed that, arriving at the middle of the channel, water molecules reverse their orientation by interacting with the asparagine residues in the two NPA motifs. This reorientation disrupts the water-water hydrogen bonds, preventing proton conduction [19]. The second mechanism, which is assessed by different authors and based on different simulations approaches [16], [20]–[22], is attributed to the existence of an electrostatic barrier located at the entrance of the AQP1 channel.

In human RBCs, the urea transporter UT-B, which contains ten transmembrane helices with intracellular N-terminus and C-terminus, carries the Kidd (Jk) blood group antigens [23]. The protein structure of a mammalian UT-B has been refined to 2.36 Å resolution [24]. Two highly conserved symmetrical structures within the channel were proposed as urea binding sites, which are separated by the so-called “Sm site” formed by two aligned threonines (Thr177 and Thr339) [24], [25]. The energy barrier observed at the Sm site corresponds to the desolvation cost of urea and plays an important role in the regulation of urea permeation [24].

In this paper, the analysis of the water permeation through UT-B and AQP1 was carried out by rapid kinetics studies using stopped-flow on RBCs with extremely rare blood group phenotypes. This approach allowed a comparison between the water permeability through UT-B and AQP1. The results from the MD simulation showed that despite the difference of structural and electrostatic characteristics, AQP1 and UT-B share comparable molecular mechanisms of water permeation.

## Materials and Methods

### Blood Samples

RBCs from two individuals of the AQP1null rare phenotype lacking AQP1 (also called Colton-null because AQP1 carries Colton antigens) donated in 1994 have been described [26], [27]. Control RBCs and RBCs from one Rhnull and three independent UT-Bnull individuals were cryopreserved since 1991 and 2003 respectively in the rare blood collection at the Centre National de Référence des Groupes Sanguins (Paris, France). Before the study that was conducted according to the ethical standards of the National Institute for Blood Transfusion (Paris, France), cryopreserved RBCs were thawed and washed three times with PBS buffer.

### Flow Cytometry Analysis

Frozen RBCs thawed and stored in stabilization solution (ID-CellStab, DiaMed) were washed three times and resuspended in PBS (DPBS, Gibco). After red cell fixation by glutaraldehyde (0.8%)/paraformaldehyde (0.025%) and permeabilisation by octylglucoside (1%), intracellular epitopes of AQP1 was detected with a FACSCanto II (BD Biosciences, San Jose, CA) using the following antibodies: mouse monoclonal anti-AQP1 (1/A5F6, Abcam, France). RBC membrane expression of RhAG was detected using the mouse monoclonal antibody LA18.18 [28]. Detection of UT-B in RBCs membranes were performed with the mouse monoclonal anti-extracellular epitope of UT-B [1]. Determinations of the RhAG and UT-B protein copy number were carried out using the Qifikit methods. Briefly, mouse monoclonal antibodies (the anti-RhAG or the anti-UT-B) were used as primary antibodies and an anti-mouse IgG conjugated to FITC was used as secondary antibody. Mouse-IgG coated calibration beads (Qifikit, Dako, Denmark) were incubated with the same fluorescent secondary antibody and were used as standard.

### RBC ghost preparation

All preparation steps, except resealing (37°C), were carried out at 4°C. Two-hundred microliters of RBCs were washed three times in PBS and resuspended in 30 ml hypotonic lysis buffer (3.5 mM K_2_SO_4_ and 10 mM HEPES pH 7.2) for 40 min on ice with gentle agitation followed by resealing for 1 h at 37°C in resealing buffer (50 mM K_2_SO_4_ and 10 mM HEPES pH 7.2) containing 1 mM MgSO_4_ and 0.15 mM pyranine, the fluorescent pH-sensitive dye (1-hydroxypyrene-3,6,8-trisulfonic acid, Sigma–Aldrich) or 15 mM ANTS (8-aminonaphthalene-1,3,6-trisulfonic acid disodium salt; Sigma-Aldrich) to measure the fluorescence quenching by D_2_O. After three washes in the resealing buffer, ghosts were kept on ice before assay in the same buffer. For proton flux, the pH of lysis and resealing buffers was fixed at 7.6.

### Water permeability measurements

The osmotic and the diffusional water permeability of RBC variants were determined by using a stopped-flow spectrophotometer (SFM74, BioLogic, France) at 15°C. Osmotic permeability was measured by mixing 80 µL of RBCs (1% hematocrit) with an equal volume of a hyperosmotic solution of mannitol. Time courses of the 90° scattered-light intensity (λ_exc_ of 530 nm) changes of red cells were measured to follow the osmotic shrinking of cells. Data from at least 4 time-courses were averaged and fitted to single exponential functions by using the Simplex procedure of the BIOKINE software (BioLogic, France).

Osmotic water permeability (Pf), in cm/s, was determined according to [29] using the following equation,
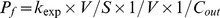
where *V/S* is the RBC volume to surface ratio, *V_W_* is the molar volume of water (18 cm^3^/mol) and *C*_out_ (mol/cm^3^) is the total concentration of extracellular solute.

Diffusional water permeability (Pd) was monitored in iso-osmotic conditions. Membrane ghosts-entrapping ANTS probe were equilibrated in resealing buffer and mixed with the same buffer prepared in D_2_O. This methodology takes advantage of the fact that the fluorescence of ANTS changes with the D_2_O∶H_2_O ratio. The excitation wavelength was 365 nm, and the emitted light was filtered with a 520-nm cut-on filter. Pd (cm/s) was calculated from a single exponential time constant (τ_ex_, exchange time) fitted to the time course of ANTS fluorescence according to Ye et al. [30], by using the following equation:
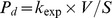
were k_exp_ (1/τ_ex_) is the exponential rate constant, V/S is the ratio of ghost volume to surface area corresponding to r/3, assuming the ghosts are spheric (r: radius of the ghosts).

### Ammonia and Proton permeability

Ammonia transport was performed by monitoring pH-sensitive fluorescence of pyranine using the stopped-flow instrument after rapid mixing of resealed ghosts with a buffer containing 10 mM (NH_4_)_2_SO_4_. P_NH3_ was calculated as described in ref [31], [32]. Concerning proton permeability (P_H_^+^), ghost loaded with pyranine were equilibrated in the resealing buffer at pH 7.6 and were mixed with same buffer containing enough H_2_SO_4_ to lower the final extracellular pH from 7.6 to 7.1. The time-course of the decrease in intracellular pH was followed on the stopped- flow instrument by monitoring the pyranine fluorescence changes. Over the pH range used, fluorescence intensities were linearly correlated to pH.

The apparent proton and NH_3_ permeability were calculated from the fluorescence time courses and the size of membrane vesicles according to the simplified equations:
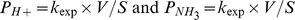



### Building human UT-B model

Homology modeling was used to determine the 3D structure of human Urea Transporter B (UT-B). The bovine structure (PDB ID: 4EZC), which shows the highest sequence identity (80%) with the target sequence, was used as the best template. Several models were constructed using *MODELLER* [33]. The final model was selected on the basis of DOPE score, which is a recommended measure to identify good models. Orientation of side-chains was determined using SCWRL4 [34] on the best model. The quality of the model was assessed with Procheck and ProQM [35], a dedicated tool to evaluate transmembrane protein model. The scores obtained for the X-ray and the model structures were very similar (Figure S1), showing that the present model can be confidently considered for further investigations.

### Molecular Dynamic (MD) Simulations

Starting from this model for a monomer, a trimer was built from the relative positioning of monomers in X-ray structure and defined as the initial structure. This structure was then minimized to remove steric clashes. The trimer was aligned along the membrane normal and embedded into a POPC lipid bilayer, hydrated with TIP3P water molecules [36] and ionized with 150 mM KCl. The full hydrated system was minimized and 500 ps of constant volume and temperature MD simulation at 300 K using Berendsen thermostat [37] with all protein heavy atoms were fixed. The whole system was simulated for 10 ns under constant pressure, and constant temperature 300 K while all protein heavy atoms were fixed. The resulting system was used as the starting point for production simulation. Note that the parameters of the system were first tested on the bovine X-ray structure, which was simulated for 100 ns. For the human sequence, two replica of 200 ns were realized on the model structure. Rmsds of each simulation are available in Figure S2. The convergence of the simulations was tested using PCA analysis. The results are provided in Figure S3.

All the simulations were performed using Gromacs software [38], with the OPLS-AA force field [39], TIP3P water and Berger lipids [40], [41] including modified parameters introduced by Monticelli [40], [41] for OPLS-AA force field.. Simulations were performed at constant temperature (300 K) using V-rescale thermostat [42] and pressure (1 bar) using a Parrinello-Rahman coupling algorithm [43]. The integration time–step was 2 fs and all bonds were constrained using P-LINCS [44]. Water molecules were kept rigid using the SETTLE algorithm. Lennard-Jones interactions were cutoff at 1.0 nm. Long-range electrostatic interactions were treated using the particle mesh Ewald approach [45] with a 1.0 nm direct space cut-off. The neighbor list was update every 10 ps and the center-of-mass motion removed at every step.

The order parameter for water inside the pore, which is defined as the average cosine of the angle between the dipole moment of water and the pore axis, was computed using the g_h2order tool of Gromacs.

### PMF calculations

The potential mean force (PMF) of water molecule as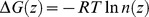, where n(z) is
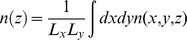
the axial distribution function [46] normalized by the TIP3P density of water along the pore of each hUT-B monomer, where 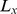 and 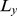 are the size of pore in 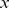 and 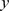 direction at each 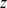 coordinate.

### Permeability coefficients

The diffusion permeation coefficient, P_d_, was determined by the quantification the equilibrium flux through the pore in MD simulations as 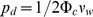 [47] where Φc is the complete bidirectional water translocations and 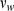 is the molar water volume. The osmotic permeation coefficient, P_f_, describes the net water flux through a pore induced by a difference in solute concentration between two compartments connected by a pore. pf was evaluated using collective diffusion method proposed by Zhu et al [47], the osmotic permeability coefficient was computed as 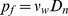 where 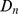 is the diffusion coefficient of the collective coordinate. This collective coordinate defined by the time depends on cumulative displacements of water molecules in the pore, normalized to the pore length.

### Electrostatic potentials

Continuum Poisson-Boltzmann electrostatic potential maps for AQP1 and human UT-B oligomers were computed using Adaptative Poisson-Boltzmann Solver (APBS) [48]. The potentials were projected on the molecular surface of the pore.

## Results

### Osmotic water permeability (P_f_) of human RBC membranes

The osmotic water permeability coefficients P_f_ were determined from a stopped-flow light-scattering analysis of RBCs exhibiting the Colton-null [2] and Kidd-null [49] phenotypes which are characterized by a total deficiency of AQP1 and UT-B, respectively (Table 1). A monoclonal antibody anti-UT-B [1] allowed the quantification of UT-B copy number on control RBCs (13.9 10^3^) corresponding to a previously determination [50].

**Table 1:** Antigen and protein expression of human RBCs.

	Control	UT-B_null_	AQP1_null_	RhAG_null_
UT-B (×10^3^ copies/RBC)	13.91±0.31	<0.8	12.22±1.35	n.t.
RhAG (×10^3^ copies/RBC)	73±12	86±10	87	<0.1
AQP1 (MFI/RBC)	1,932±296	2,523±697	53	2,525±441

Values indicate the copy number of membrane proteins per red cell (×10^3^), except for AQP1 (anti-Colton antigen), which was measured as mean of fluorescence intensity. n.t.: not tested. MFI: Mean of Fluorescence Intensity.

**Figure 1:**
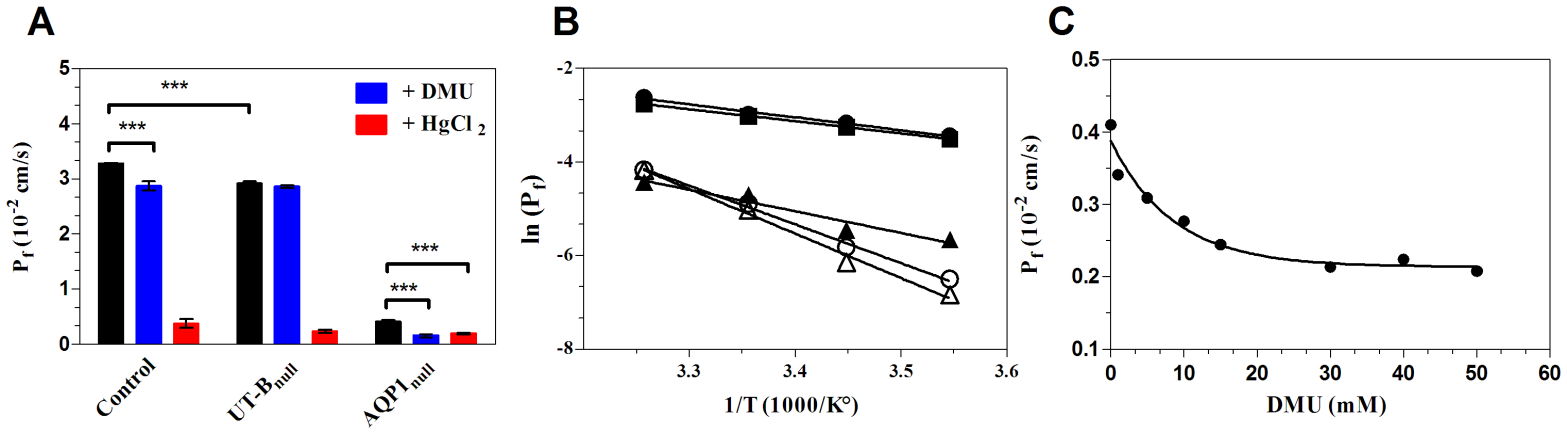
Osmotic water permeability of RBCs. (A) Coefficients of osmotic water permeability (P_f_) at 15°C in the absence or the presence of inhibitors (Blue, 15 mM DMU; red, 0.5 mM HgCl_2_). Three experiments for each individual were averaged and the means of the rate constants (k, s^−1^) for 3 controls, 3 UT-B_null_ and 2 AQP1_null_ were reported (± SD). Statistical significances between the P_f_ of DMU and/or HgCl_2_-treated versus untreated RBCs (control and AQP1_null_) were determined by paired t tests. Statistical significances between the P_f_ of control versus UT-B_null_ RBCs were determined by unpaired t tests. *** indicates a significant difference (p<0.0001). (B) Arrhenius activation energies Ea (which are related to the slope of the plot) of osmotic water permeation across RBC membranes (filled circles, normal; squares, UT-B_null_; filled triangles, AQP1_null_; open triangles, AQP1_null_+HgCl_2_; open circles, AQP1_null_+DMU). (C) Coefficients of osmotic water permeability for control RBCs incubated with increasing concentrations of DMU and submitted to a 400 mosm/kgH_2_O mannitol osmotic gradient of at 15°C.

The P_f_ was determined at 15°C for each RBC variant. As expected, the absence of AQP1 in AQP1_null_ individuals causes a strong decrease of P_f_ (Figure 1A). More interestingly, the measurement of water permeability in UT-B_null_ RBCs shows a small (12±2%) but significant reduction of P_f_ (Figure 1A). The Arrhenius activation energy (Ea) of P_f_ was measured (Figure 1B) for UT-B_null_ RBCs (5.9 kcal/mol) as well as for normal control red cells (5.1 kcal/mol). These low Ea can be explained by the presence of AQP1, either in the absence of UT-B or with an AQP1/UT-B copy number ratio close to 10 derived from published data [8], [51] for AQP1 and our present results for UT-B. This contributes to the low temperature-dependent water movements in both cases. As expected, the Ea for the AQP1_null_ RBCs was higher (9.5 kcal/mol).

Since inhibition of water permeability of RBCs by mercurial salts was taken as evidence that protein water pores must exist [52], we have determined the Ea for AQP1_null_ RBCs in the presence of HgCl_2_ (0.5 mM). Interestingly, the Ea was increased to a value of 19.2 kcal/mol (Figure 1BC), which is similar to the value obtained for water osmotic permeability across lipid bilayers [8]. [2] Addition of 0.5 mM HgCl_2_ to all RBC samples significantly decreased the P_f_ values and resulted in very similar residual water permeabilities regardless the phenotype of the erythrocytes (Figure 1A). These results indicate that AQP1 is not the only pathway for water transport in human RBCs and that at least a second mercury-sensitive protein might assume the residual water permeability in AQP1_null_ RBCs, in agreement with previous works [2].This also suggests that UT-B protein and/or AQP3 proteins could be, like AQP1, a mercury-sensitive water channel. However, previous studies [7], [8], [53] showed that UT-B was not sensitive to mercury chloride.

The urea analogue dimethylurea (DMU) was previously used as a urea inhibitor in structural studies of the bacterial urea transporter dv-UT [25]. In the present study, it was employed in order to investigate its ability to inhibit the water flux through UT-B. AQP1_null_ RBCs submitted to a 400 mosmol/kgH_2_O osmotic gradient of mannitol were incubated with increasing concentration of DMU (Figure 1C). This led to a progressive decrease of P_f_ values, indicating that DMU prevents the passage of water molecules through the pore of UT-B. Interestingly, the Ea for AQP1_null_ RBCs in the presence of DMU (15 mM) was increased (16.5 kcal/mol), like in the presence of HgCl_2_. Moreover, the addition of 15 mM DMU which corresponds to maximal inhibition, decreased the P_f_ value of normal erythrocytes to 2.86±0.09 10^−2^ cm/s, which is similar to the P_f_ value measured for UT-B_null_ RBCs, incubated or not with DMU (Figure 1A), indicating no effect of DMU on AQP3 conductance. This result shows the specific and complete inhibition of water transport through human UT-B by this urea analogue. In AQP1_null_ RBCs, the same inhibiting effect of DMU and HgCl_2_ was observed on the P_f_ value, which is decreased by 56% when compared to the P_f_ of these untreated RBCs (Figure 1A). Therefore, the remaining water permeability in AQP1_null_ RBCs previously treated with DMU or HgCl_2_ can be attributed to the DMU-insensitive AQP3, the mercury-insensitive UT-B and/or the lipid bilayer.

Taken together, our results demonstrate that, besides the major water channel AQP1, UT-B contributes to about 10% of the total osmotic water transport through human RBCs. To determine and compare the unit permeabilities of AQP1 and UT-B, the copy number per erythrocyte of both proteins was necessary. While pf_unit_ for UT-B (Table 2) was deduced from our determination of the UT-B copy number (Table 1), a mean of pf_unit_ for AQP1 (Table 2) was calculated from a range of published AQP1 copy number values [8], [51]. Curiously, the single-channel osmotic permeability coefficients (pf_unit_) for AQP1 (23.5 10^−14^ cm^3^/s) and UT-B (25.8 10^−14^ cm^3^/s) were of the same order of magnitude.

**Table 2:** UT-B and AQP1 unit permeabilities deduced from rate constants and permeabilities to water (pf and pd), to proton and to ammonia of human RBC variants.

			Variants		Proteins	
Substrates		Control	UT-B_null_	AQP1_null_	UT-B†	AQP1††
H_2_O osmotic						
	*k, s^−1^*	6.15±0.02	5.47±0.07	0.76±0.05	-	-
	*P_f_,10^−2^ cm/s*	3.27±0.01	2.91±0.04	0.41±0.03	-	-
	*p_funit_,10^−14^ cm^3^/s*	-	-	-	25.8±0.81	23.5 (19.4–27.7)
H_2_O diffusional						
	*k, s^−1^*	42.51±0.73	37.02±1.41	11.27±0.44	-	-
	*P_d_,10^−3^ cm/s*	2.77±0.04	2.41±0.09	0.73±0.02	-	-
	*p_dunit_,10^−14^ cm^3^/s*	-	-	-	3.51±0.11	1.67 (1.38–1.97)
H^+^						
	*k, s^−1^*	0.55±0.06	0.53±0.03	0.49±0.02	-	-
	*P, 10^−5^ cm/s*	3.36±0.17	3.15±0.08	3.01±0.17	-	-
	*p_unit_,10^−17^ cm^3^/s*	-	-	-	∼0	∼0
NH_3_						
	*k, s^−1^*	2.58±0.12	2.28±0.1	1.81±0.2	-	-
	*P,10^−4^ cm/s*	1.53±0.09	1.35±0.29	1.05±0.05	-	-
	*p_unit_,10^−16^ cm^3^/s*	-	-	-	∼0	1.91–2.54

† UT-B unit permeabilities calculated from the number of UT-B copy per RBC which is described in table 1.

†† Means and ranges of AQP1 unit permeabilities calculated from the published values of AQP1 copy number (150 000–200 000) [8], [51]. Experiments performed at 15°C.

### Diffusional water permeability (P_d_) of human RBC membranes

**Figure 2:**
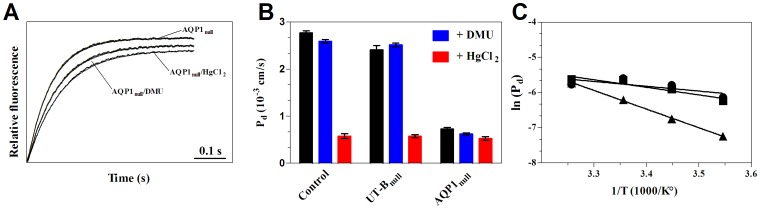
Diffusional water permeability of RBCs. (A) Time course of the diffusional water permeability at 15°C in ghost from AQP1_null_ phenotype, in the presence or absence of HgCl_2_ (10 mM) or DMU (0.5 mM). Smooth lines are exponential fits to the data using the simplex procedure of the Biokine (Bio-logic), providing rate constant of water permeability k (s^−1^). (B) Coefficients of diffusional water permeability (P_d_) at 15°C in the absence or the presence of inhibitors (Blue, DMU; red, HgCl_2_). Three experiments for each individual were averaged and the means of the rate constants k (s^−1^) for 3 controls, 3 UT-B_null_ and AQP1_null_ were reported (± SD). (C) Arrhenius activation energies (which correspond to the slope of the plot) of diffusional water permeation across RBC membranes (circles, control; squares, UT-B_null_; triangles, AQP1_null_).

The significant osmotic water permeability of UT-B suggests the existence of a continuous aqueous channel through this protein. In order to further define the mechanisms of transport, diffusional water permeability was measured by fluorescence changes of a probe (ANTS) in a D_2_O medium. Figure 2A shows the time-course of water diffusion across AQP1_null_ RBCs. Addition of DMU or HgCl_2_ to AQP1_null_ RBCs resulted in slower kinetics (Figure 2A), which corresponds, as compared to the untreated AQP1_null_ RBCs, to a decrease in P_d_ values of 13 and 25%, respectively (Figure 2B).

Similarly, consistent with the expression levels of AQP1 and UT-B channels, AQP1 and UT-B deficiencies caused a decrease in P_d_ values of 75 and 10%, respectively (Figure 2B). Finally, compared to the Arrhenius activation energy of P_d_ obtained for normal RBCs (4.5 kcal/mol), the Ea of free diffusion of water in the absence of either AQP1 or UT-B was higher (10.4 and 5.2 kcal/mol, respectively) (Figure 2C). These results show that water molecules diffuse inside a continuous aqueous pathway not only through AQP1 but also through UT-B.

Taking into account the expression levels of both proteins (Table 1), the diffusional water permeability through a single channel of UT-B is 3.51 10^−14^ cm^3^/s versus 1.67 10^−14^ cm^3^/s for AQP. The ratio of the osmotic to the diffusive permeability coefficients allowed the approximate determination of the number of water molecules ((P_f_/P_d_)−1) that are involved in the water transport through UT-B and AQP1, which is 6 and 13, respectively.

### Proton and ammonia permeability

In order to compare the selectivity of the water-channels AQP1 and UT-B, proton and ammonia permeability were measured in ghosts from RBC variants.

**Figure 3:**
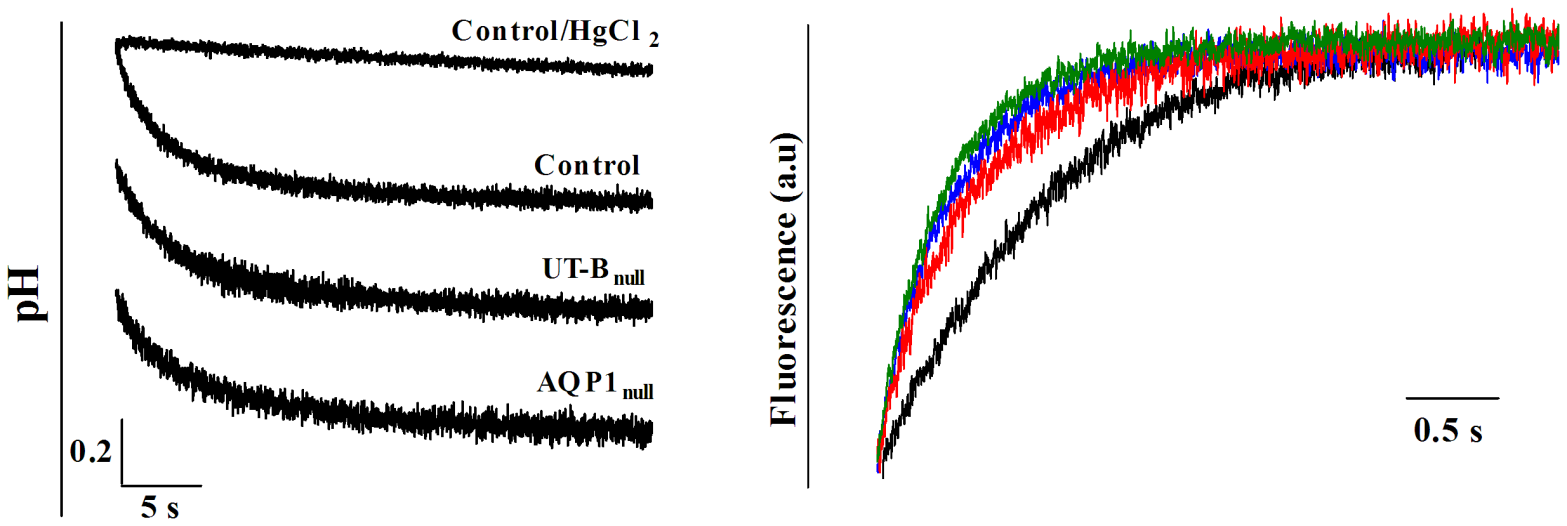
Comparison of Proton and ammonia transports in ghost from RBC variants. (A) Time course of intracellular pH decrease in ghosts subjected to pH gradient from 7.1 to 7.6. (B) Time course of fluorescence changes of ghosts derived from control (green), UT-B_null_ (blue), AQP1_null_ (red), RhAG_null_ (black) RBCs and subjected to 10 mM ammonium inwardly-directed gradient followed by stopped-flow analysis.

Representative time-courses of pyranine fluorescence changes corresponding to proton conductance measurements through the membrane of control, UT-B_null_ and AQP1_null_ RBCs are reported in Figure 3A. This shows the decrease of intracellular pH of ghosts submitted to an inwardly-directed proton gradient. The fluorescence decrease followed the sum of two exponentials revealing two distinct kinetics of the proton conductance: a rapid and a slower one. After addition of HgCl_2_ to ghosts derived from control red cells, the rapid phase of acidification totally disappeared, suggesting that the first phase might be facilitated by a mercury-sensitive channel. However, as expected in the absence of treatment, no difference exists between control and AQP1_null_ RBCs, which is in agreement with a proton impermeability, a mechanism that has been largely studied regarding AQP1 [54] and which corresponds to the exclusion of protons from the pore of the channel. Similarly, the absence of UT-B from UT-B_null_ RBCs does not significantly affect the kinetic rate constant (3.15±0.08 versus 3.36±0.17 10^−5^ cm/s at 15°C) of proton uptake, suggesting that the urea channel can also be considered as impermeable to protons (Table 2). These results indicate that, despite their mercury sensitivity regarding water transport, neither UT-B nor AQP1 can account for the effect of HgCl_2_ on proton movements through the red cell membrane.

Since ammonia has a molar volume, dimension and dipole moment similar to that of water, membrane permeabilities to ammonia were also investigated in the different variants. As shown in Figure 3B, P_(NH3)_ in Rh_null_ RBCs (0.72±0.12 10^−4^ cm/s) is twice lower than in normal RBCs (1.53±0.09 10^−4^ cm/s), confirming that the Rh-associated glycoprotein (RhAG) mediates facilitated transport of NH_3_ into human RBCs [32]. We also found that AQP1_null_ RBCs, with normal expression levels of RhAG (Table 1), showed a significant (p<0.05) reduction of P_(NH3)_, showing that AQP1 might be slightly permeable to NH_3_, as suggested by Nakhoul et al. [55]. We found that UT-B_null_ RBCs, however, showed no significant (p>0.05) reduction of the alkalinization rate (1.35±0.29 10^−4^ cm/s) compared to the control (1.53±0.09 10^−4^ cm/s) (Table 2). This result indicates that UT-B seems not to be involved in NH_3_ transport in our experimental conditions, in contrast to recently published results [56]. Although RBC variants used in the present study gave evidence of water permeation through UT-B, the measurement of ammonia transport across this channel might be limited by the high RBC lipidic ammonia diffusion and/or by the weak NH_3_ unit permeability of UT-B, compared to that of water.

### Mechanism of water permeation through UT-B

**Figure 4:**
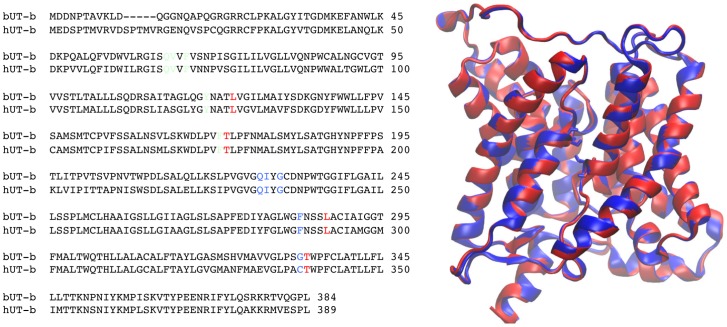
Alignment and superposition between bUT-B and hUT-B. Alignment between bovine and human UT-B shows 80% of sequence identity. Superimposition between human model (red) and bovine structure (blue) on Cα is less than 1 Å.

**Figure 5:**
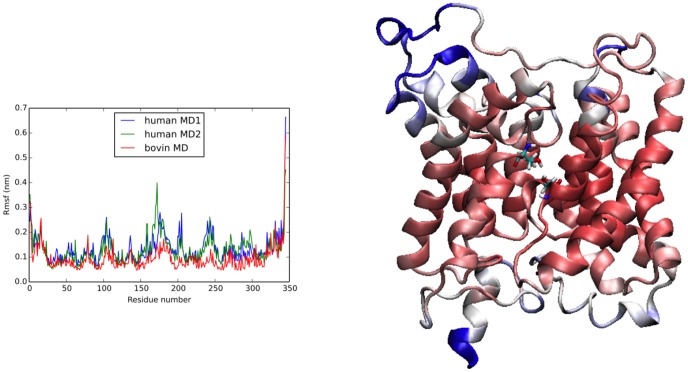
Cα fluctuations computed from each simulation. (a) Root Mean Square Fluctuation (RMSF), RMSF of simulations on human model (blue and green) and bovine structure in red. (b) Same information projected on the 3D structure. In red, residues exhibiting low fluctuations, and in blue, residues with high fluctuations.

To provide a structural and dynamic description of water permeation in human UT-B and determine the process engaged in water molecules crossing human channel, we performed two MD simulations on a 3D model obtained by homology modeling from the bovine UT-B (bUT-B) structure [24]. The sequence alignment (Figure 4) shows a high degree of conservation, with few gaps, located mainly in loop regions. During simulations, the largest fluctuations are located in loop regions (Figure 5). As expected, transmembrane helices exhibit small fluctuations except in a bent and less conserved region located between residues 319 to 327 in helix 8 in simulation of human model and bovine structure. Interestingly this region is associated with large B-factors in the bovine structure. The motions in this region directly impact the positioning of the residues in the extracellular vestibule, close to the entrance. Indeed, as dynamics progresses, the distance between Cα-Thr177 and Cα-Thr339 rapidly increases from 0.7 nm to reach ∼0.9 nm. Yet, the H-bond between Thr339 and Asn181 is maintained all along the simulation. A 200 ns-MD replica showed a similar behavior.

Even though precise delimitation of the pore remains questionable, we chose for defining the pore, similar zones as for the bUT-B structure and used the same nomenclature as in Levin et al [24] *i.e* three regions S_0_, S_m_ and S_i_ indicated in Figure 4. The two conserved residues, Thr177 and Thr339, shown to be important for urea transport [24], are included in this pore region. For Thr339, only backbone atoms are accessible to the pore lumen while side chain atoms are exposed for Thr177 (Figure 5).

**Figure 6:**
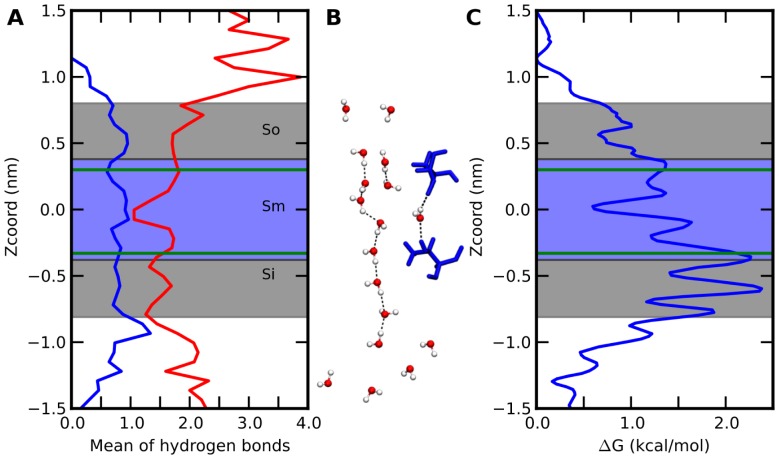
Hydrogen bonds and potential of mean force of water in the pore. (A) The average number of hydrogen bonds over the MD simulation between water and, in blue, the protein and, in red, the water molecules. (B) Snapshot of water molecules' organization in the lumen of the pore, with the hydrogen bond network. Thr177 and Thr339 are displayed in blue. (C) Potential of Mean Force of a water molecule along the pore calculated from the water density. Green lines indicate the average z-coordinate of CG atoms for Thr177 and 339, over the three subunits. Residues in stick define sub-site along pore axis.

On the time scale of simulations (200 ns), we observed the passive diffusion of water molecules in UT-B along the same pore identified for the transport of urea, the natural substrate (Figure 6). No transport occurred through the central pore of UT-B trimer. The average time required for a water molecule to cross the pore delimited by S_m_ to S_i_ regions was ∼0.5 ns. In comparison, the number of events observed for the three monomers is 257 for 200 ns, which would give an apparent rate of 0.43 water/monomer/ns. The permeation parameters p_d_ and p_f_ computed using the methodology defined by Zhu et al [47] were 2.75±0.92 10^−14^ cm^3^/s and 16.3±3.0 10^−14^ cm^3^/s, respectively (Table 3). Evaluation of permeation parameters on bovine form gave similar results (1.16 10^−14^ for p_d_ and 11.0 10^−14^ for p_f_).

**Table 3:** Water conduction through UT-B and AQP1.

	Simulation					Experimental		
	p_f_	p_d_	N	O	L (Å)	p_f_	p_d_	N
UT-B	16.3	2.75	4.9	5	14	25.8	3.5	6.3
AQP1	7.1–10.3*	0.6–0.8*	10–12*	6–9*	18	23.5	1.6	13.1

Single-channel Osmotic (Pf) and diffusional (Pd) water permeability are given in 10^−14^ cm^3^·s^−1^. O and L correspond to the luminal water occupancy and lumen length, respectively. N corresponds to the number of water molecules in the lumen, determined by Pf/Pd −1.

* Values described by [47], [60].

To better understand the transport mechanism in UT-B, we examined the average number of hydrogen bonds *per* water molecule passing through the UT-B pore (Figure 6A). In the external vestibule (S_o_) (0.75 nm<Z<0.4 nm), the water molecules are stabilized by a total of ∼3 hydrogen bonds with protein residues and preferentially with water molecules. When approaching the S_m_ region of the pore (0.4 nm<Z<−0.4 nm), in the region located between Thr177 and Thr339, the number of water–water (W-W) hydrogen bonds decreases significantly while the number of hydrogen bonds with the residues lining the pore increases concomitantly. The water molecule crossing the region located between Thr177 and Thr339 maintained 1.5 W-W hydrogen bonds on average. After this zone (S_i_, −0.4 nm<Z<−0.75 nm), the water molecule recovers water and protein partners to restore the stabilizing effect of 3 hydrogen bonds. It is noteworthy that beside mobile water molecules crossing the pore rather rapidly, water molecules with long residence time (∼20 ns) appeared to be trapped in a bridge connecting the two Thr residues aforementioned (see Figure 6B). To further characterize the water transport mechanism through UT-B, we calculated a potential of mean force (PMF) of water in human UT-B from water density (Figure 6C). In the S_0_ region, the water molecule first encounters a small barrier (0.6 nm from the center) followed by a local minimum at 0.5 nm from the center. In the S_m_ region, a small minimum close to Thr339 is followed by a deeper minimum located at the center. The passive diffusion of water indicates that the energy barriers encountered along the pore can be crossed by thermal fluctuations. The PMF of water recently calculated for bUT-B [56] shows very similar results for the two species. Height barriers agree within 0.5 kcal/mol despite the different methodologies used.

**Figure 7:**
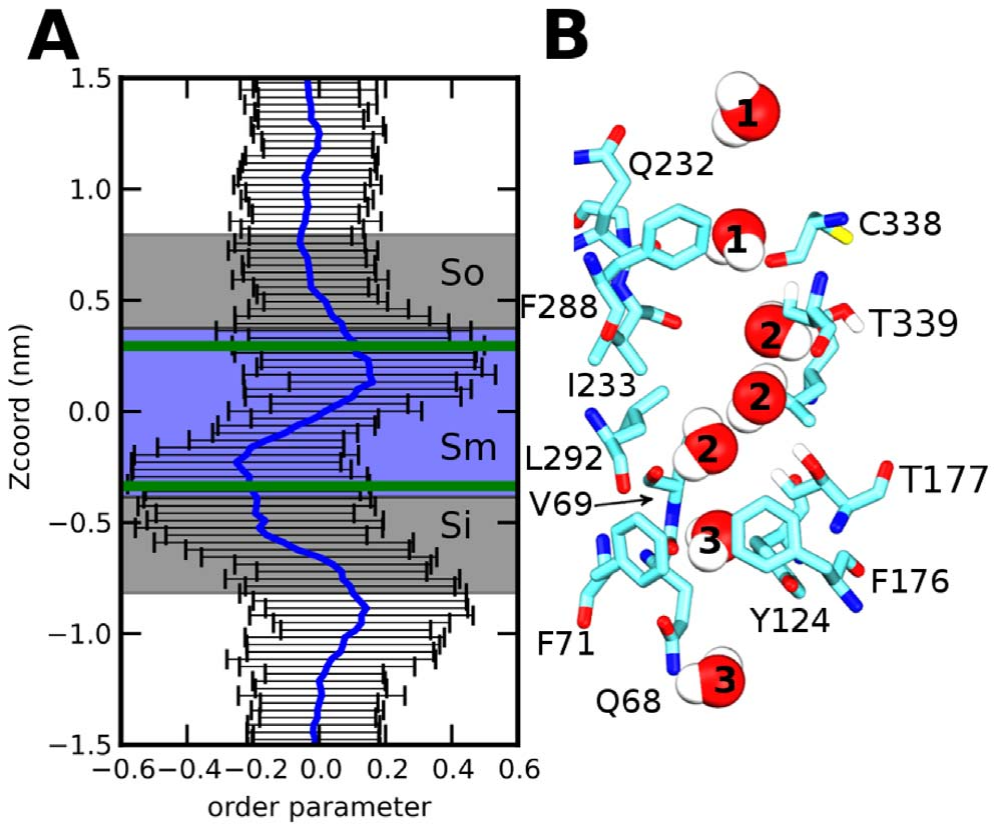
Orientation of water molecules through the pore. (A) Order parameter of water molecule through the pore with standard deviation (B) Different positions adopted by a mobile water molecule, crossing the pore. Green lines, same definition as Fig. 6.

The dipolar moment orientation of the diffusive water molecule with respect to the pore axis shows significant variations (Figure 7A). In the large extracellular vestibule, the water molecule can rotate freely as exemplified by a dipolar moment order close to 0. In the pore, in the vicinity of Thr339, the water molecule adopts a preferred orientation (conformation 1, Figure 7B, upper panel). Then, rapidly the water molecule rotates to adopt an orientation roughly perpendicular to the previous one (conformation 2, Figure 7B, middle panel). This reorientation is correlated to the formation of H-bond with Thr177 residue (Figure S4). Then, the water reorients parallel to the channel axis before reaching the large intracellular vestibule, (conformation 3, Figure 7B. bottom panel) where no preferred orientation is observed.

## Discussion

The present study uses a new experimental approach, measuring the osmotic and diffusional water permeabilities across human RBC membranes from natural variants characterized by deficiencies of UT-B or AQP1. Since tools allowing the measurement of the expression level of human UT-B and AQP1 are easily available, in contrast to that of mice and other species, the permeability units for each channel can be precisely determined and compared (Table 2). In *Xenopus* oocytes, the precise density of recombinant UT-B, corresponding or not to physiological levels, seemed to be critical for its ability to transport water [7], [53]. However, in this heterologous system, the threshold of physiological significance remains complex to establish.

The significant decrease of P_f_ and P_d_ values observed in UT-B_null_ RBC compared to controls clearly demonstrates the definite contribution of UT-B to water permeability of human RBCs. In addition, the use of DMU, known to inhibit urea [57], resulted in a specific inhibition of water permeability in the absence of AQP1 and resulted in no effect when UT-B is absent. This data is consistent with a significant intrinsic water permeability of UT-B and with an inhibition mechanism of DMU by blockage of the UT-B pore [25].

The P_f_/P_d_ ratio for UT-B calculated from experimental data and MD simulations gave similar values (Table 3) and are both in agreement with that of a water channel [58]. In the pore lumen, the water occupancy observed in MD simulations is 5 molecules, which correspond to (P_f_/P_d_) −1 (Table 3). This result and an effective pore radius of 2.21 Å are consistent with a chain of water molecules organized as a “single file”, as previously described for AQP1 [59], [60].

### Comparison of water conduction through UT-B and AQP1

Despite the high difference in overall architecture between AQP1 and UT-B, they present similar ranges of permeabilities, selectivities, and other dynamic characteristics. AQP1 and UT-B have similar values of P_f_ but the P_d_ value of UT-B is found to exceed that of AQP1 (2–4 fold). In both channels, water occurs in a highly correlated single file configuration, with experimental P_f_/P_d_ ratios equal to 7 and 14 for UT-B and AQP1, respectively [47], [61] (Table 3). The water occupancy inside the channel lumen of UT-B deduced from simulation data, is lower than that of AQP1 (between 6 and 9).These differences are in agreement with the length of the lumen in each channel (18 Å for AQP1 and 14 Å for UT-B).

It is of interest to observe the similar reorientation of water dipoles within the pore of UT-B and AQPs. As shown in previous simulations and Fig. 7B, water dipole reverses direction when passing across the NPA in AQP1 and S_m_ regions in UT-B. In the case of AQP1, it was shown that the orientation of water dipoles is controlled by hydrogen bonding with two Asn in the NPA motifs and the macro-dipoles formed by the hemi-helices [15]. Concerning UT-B, water reorientation observed in the S_m_ region correlates to the formation of hydrogen-bonds with Thr177 and Thr339. Note that water reorientation is one of the mechanisms for preventing proton transduction by the hop-and-turn Grotthuss relay mechanism. The other one is attributed to the existence of a large electrostatic entrance barrier in AQP1 [62].

**Figure 8:**
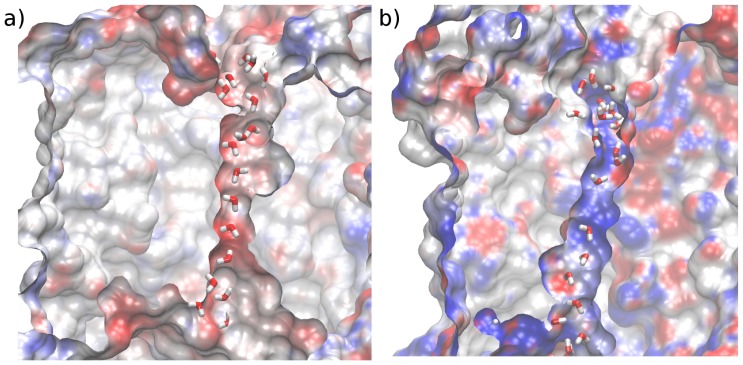
Electrostatic potential on the surface of UT-B (a) and AQP1 (b). View along the pore with water molecules located in the pore. Electrostatic potential defined between −10 (red) and 10 (blue) KT/e.

Interestingly, the electrostatic potential in the pore lumen differs between AQP1 and UT-B. As exemplified in Fig. 8, the electrostatic potential along the AQP1 channel interior surface is mainly positive, except in a small zone close to the NPA motif. In contrast, the channel interior surface of UT-B is mainly negative. This negative potential may trap positive chemical species. Hence, proton exclusion observed experimentally in UT-B requires different mechanisms than those suggested for AQP1. In any case, this might impact the selectivity properties of both channels for charged species.

### Comparisons of water and urea transport through UT-B

Water molecules were found to be intimate participants in the conduction of urea, and both water and urea molecules are observed through the UT-B channel [24]. The comparison with PMF urea profile of the bUT-B recently described by Levin et al. [24] shows interesting similarities, namely a comparable barrier for urea close to the S_0_ site, a local minimum at 0.5 nm from the center, deeper for urea, followed by the two minima in the S_m_ region. The most notable difference lies in the barrier heights, which are 2-fold smaller for water (2.5 kcal/mol) (Figure 6C) than for urea (5.0 kcal/mol).

Whereas a large part of the energetic barrier for urea can be attributed to the desolvation cost in the S_m_ region [24], water permeation through the same pore is only controlled by the hydrogen-bonding interactions among waters and with the luminal residues. Together with different sizes of water and urea, this observation is in agreement with a transport turnover rate of UT-B slower for urea (2–6×10^6^ molecules.channel^−1^.s^−1^ [50]) than for water (4.3×10^8^ molecules.channel^−1^.s^−1^, as determined here by simulation). Furthermore, the two threonines that are located in the S_m_ region and that were shown to be critical for urea desolvation [24], are also involved in water conduction through the UT-B pore, as shown here. This observation opens the question of a potential competition between water and urea to cross the channel.

Interestingly, the mammalian urea transporters UT-A2 and UT-A3, which have been shown to be impermeable to water, exhibit urea transport rates that are 100-fold less than that of UT-B [63], [64]. It is surprising that UT-As are not efficient for water transport, since a high conservation between the two families of urea transporters (UT-A and UT-B) can be observed regarding the critical residues in the pore [24]. Structural analysis and MD simulation of UT-As should be informative on hydrogen exchange between water and urea through these channels.

In addition, it has been shown that urea transport through UT-B is modulated by osmotic stress [24]. However, these studies are from oocytes which are characterized by an extremely weak water permeability, consequent to the absence of AQP [65]. In order to test the role of AQP1 in the osmoregulation process of urea transport through UT-B, it will be interesting to perform P_urea_ experiments on variant RBCs, in which AQP1 and UT-B are co-expressed or not, subjected to different osmotic conditions.

In human RBCs, abundant AQP1 (∼2 10^5^ copies/cell) assume the major water movements (84%) across the membrane in response to osmotic stresses [66], whereas 14 000 copies of UT-B can only moderately contribute to this function. However, one may speculate that aqueous pore through UT-B could play an important role in its highly efficient urea transport function.

In the present paper, a functional study of human RBC variants and MD simulations clearly demonstrate that urea and water share the same pathway through the pore of UT-B. These new insights raise the question of whether urea and water are able to compete with one another in order to affect each other's permeability. This issue will be further studied by urea uptake experiments on human AQP1-deficient RBCs, thus contributing to a better understanding of the role of the UT-B in water homeostasis.

## Supporting Information

Figure S1 **ProQM scores along the UT-B sequence.**
(TIF)

Figure S2 **Root Mean Square Deviation (RMSD) on Cα atom versus time.** RMSD on each simulation of human model in blue and in green, in red simulation of Bovine structure.
(TIF)

Figure S3 **Inner product matrix on 10 first eigenvector of PCA.** Matrix on transmembrane region of UT-B trimer (left). Matrix on transmembrane of a monomer (right).
(TIF)

Figure S4 **Hydrogen bonds between water molecules depicted in** **Figure 7b** **and pore residues.** The figure is given separately for clarity.
(TIF)

File S1 **Construction and evaluation of structural model for the human sequence and simulation convergence.**
(PDF)
