# Supplementary figures and images for: Energetic and Molecular Water Permeation Mechanisms of the Human Red Blood Cell Urea Transporter B
Source: PLoS One. 2013 Dec 20;8(12):e82338. doi: 10.1371/journal.pone.0082338 (PMC3869693; doi:10.1371/journal.pone.0082338)

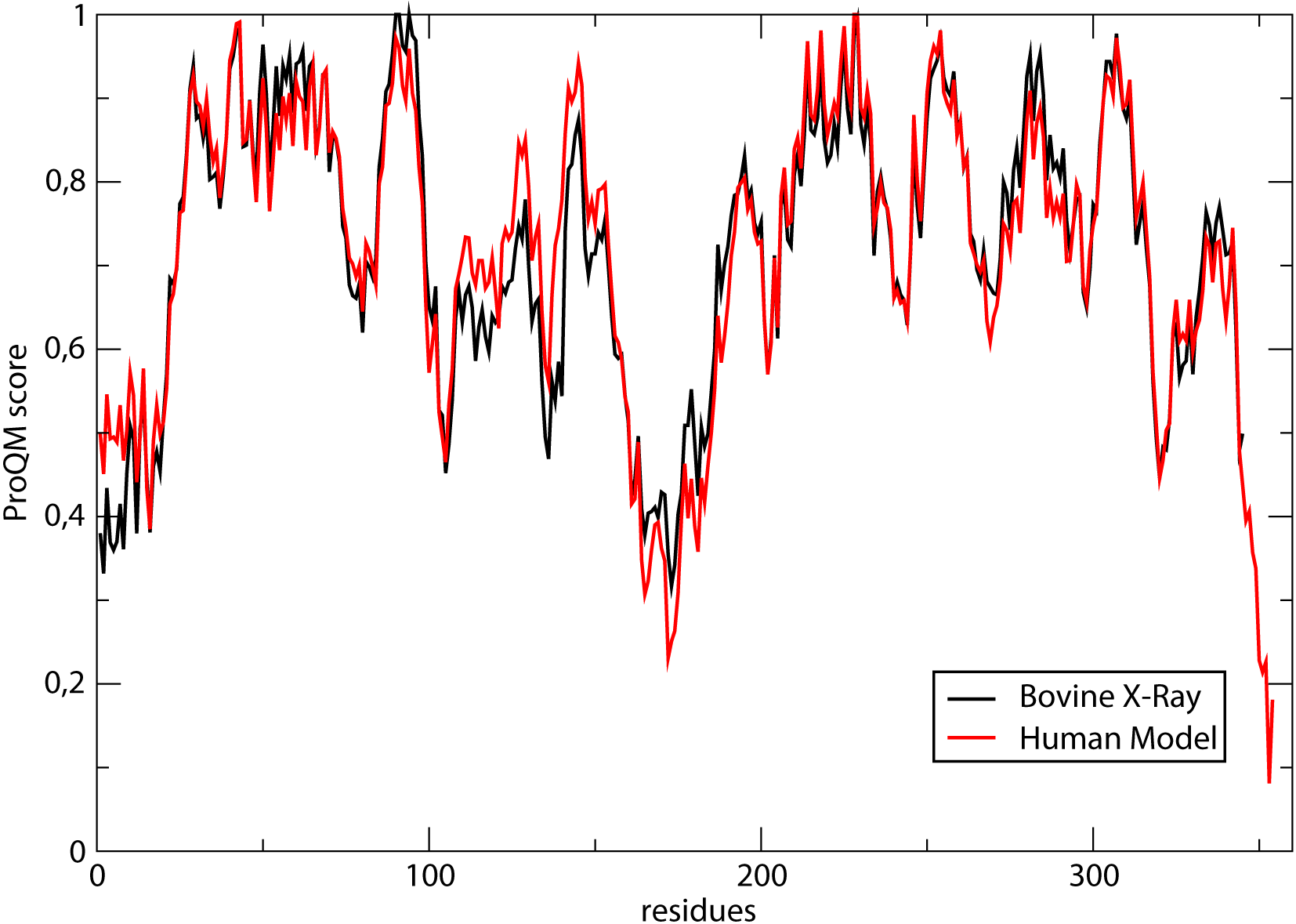

Supplement: Figure S1 — ProQM scores along the UT-B sequence. (TIF) [file pone.0082338.s001.tif]

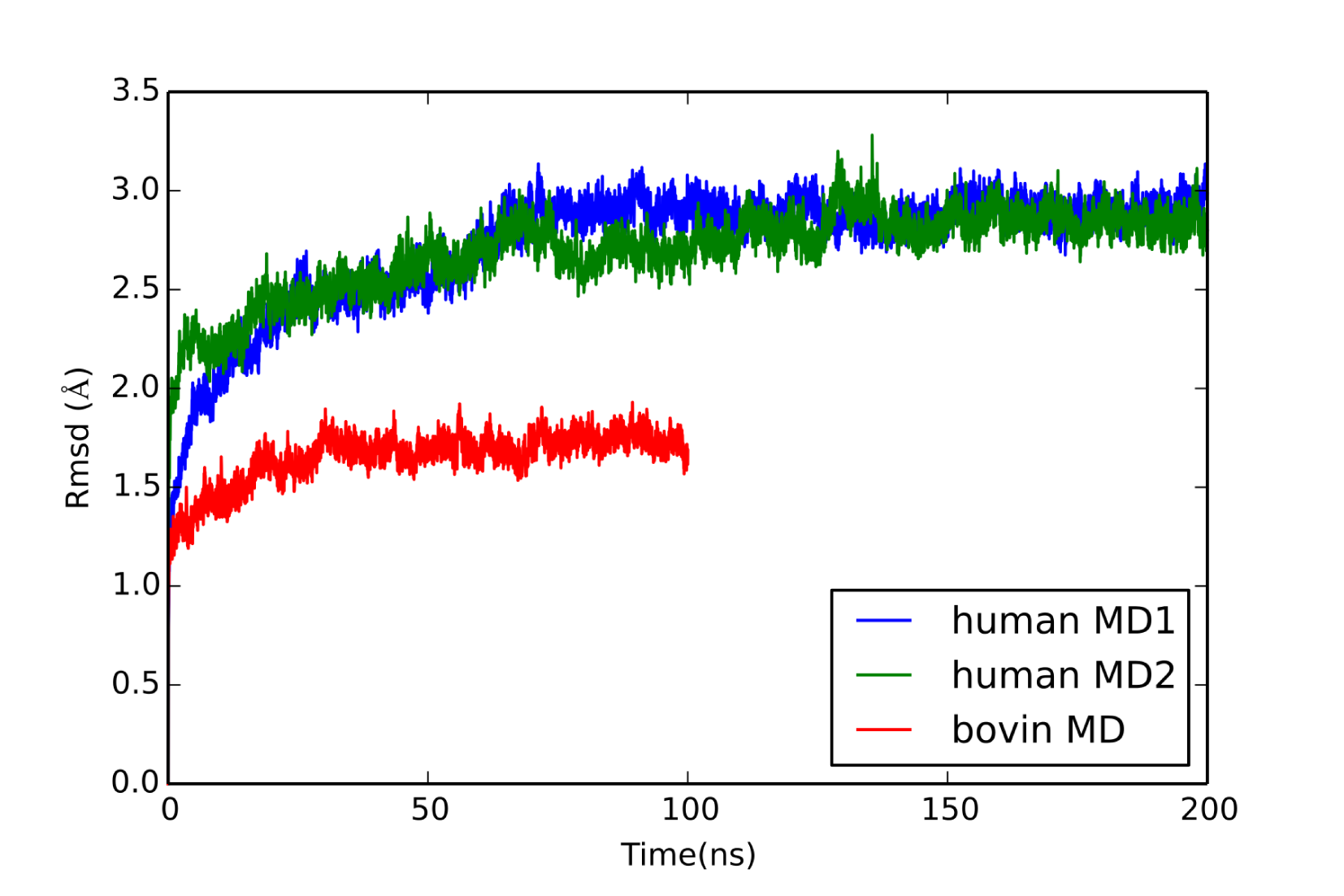

Supplement: Figure S2 — Root Mean Square Deviation (RMSD) on Cα atom versus time. RMSD on each simulation of human model in blue and in green, in red simulation of Bovine structure. (TIF) [file pone.0082338.s002.tif]

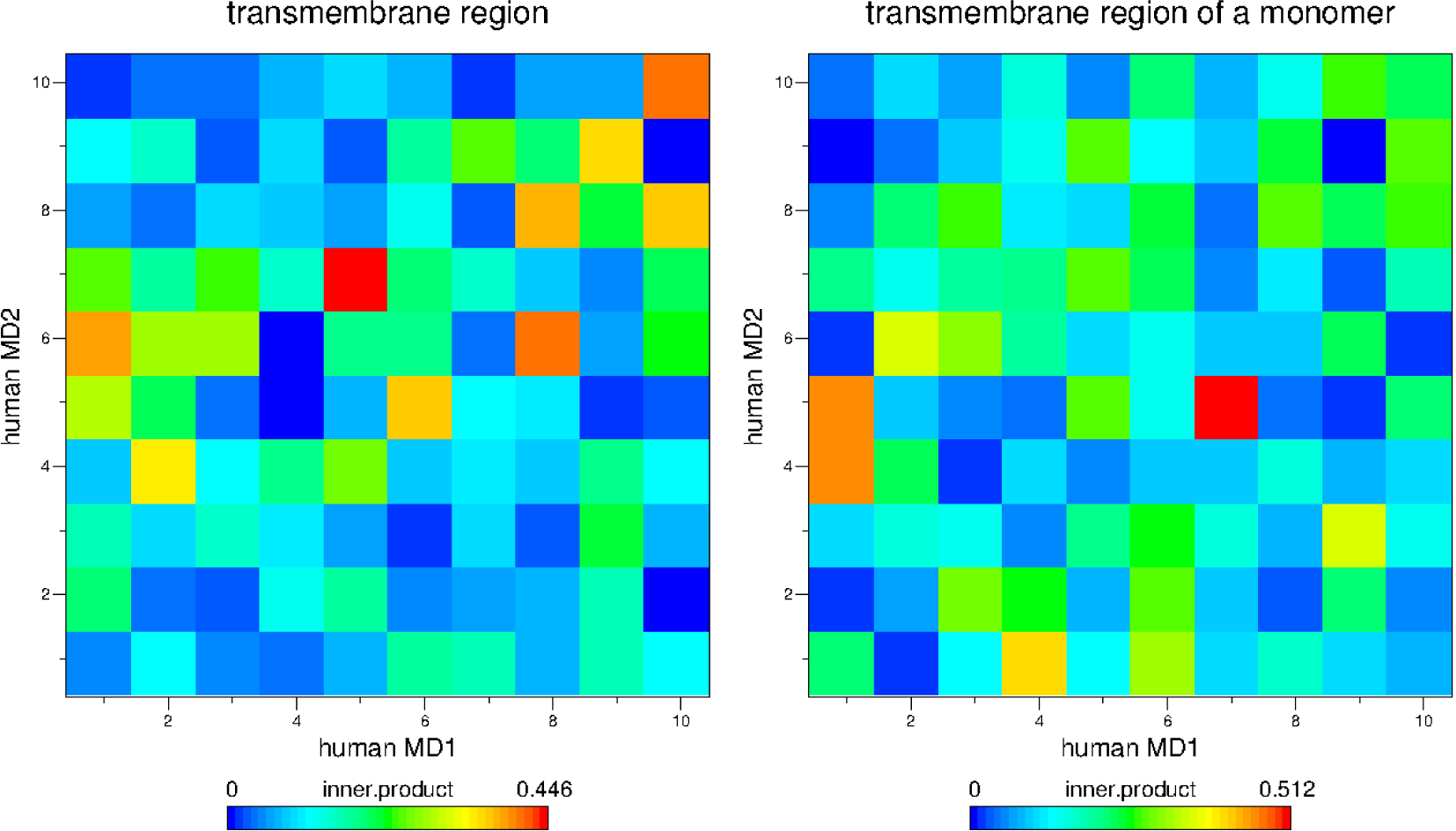

Supplement: Figure S3 — Inner product matrix on 10 first eigenvector of PCA. Matrix on transmembrane region of UT-B trimer (left). Matrix on transmembrane of a monomer (right). (TIF) [file pone.0082338.s003.tif]

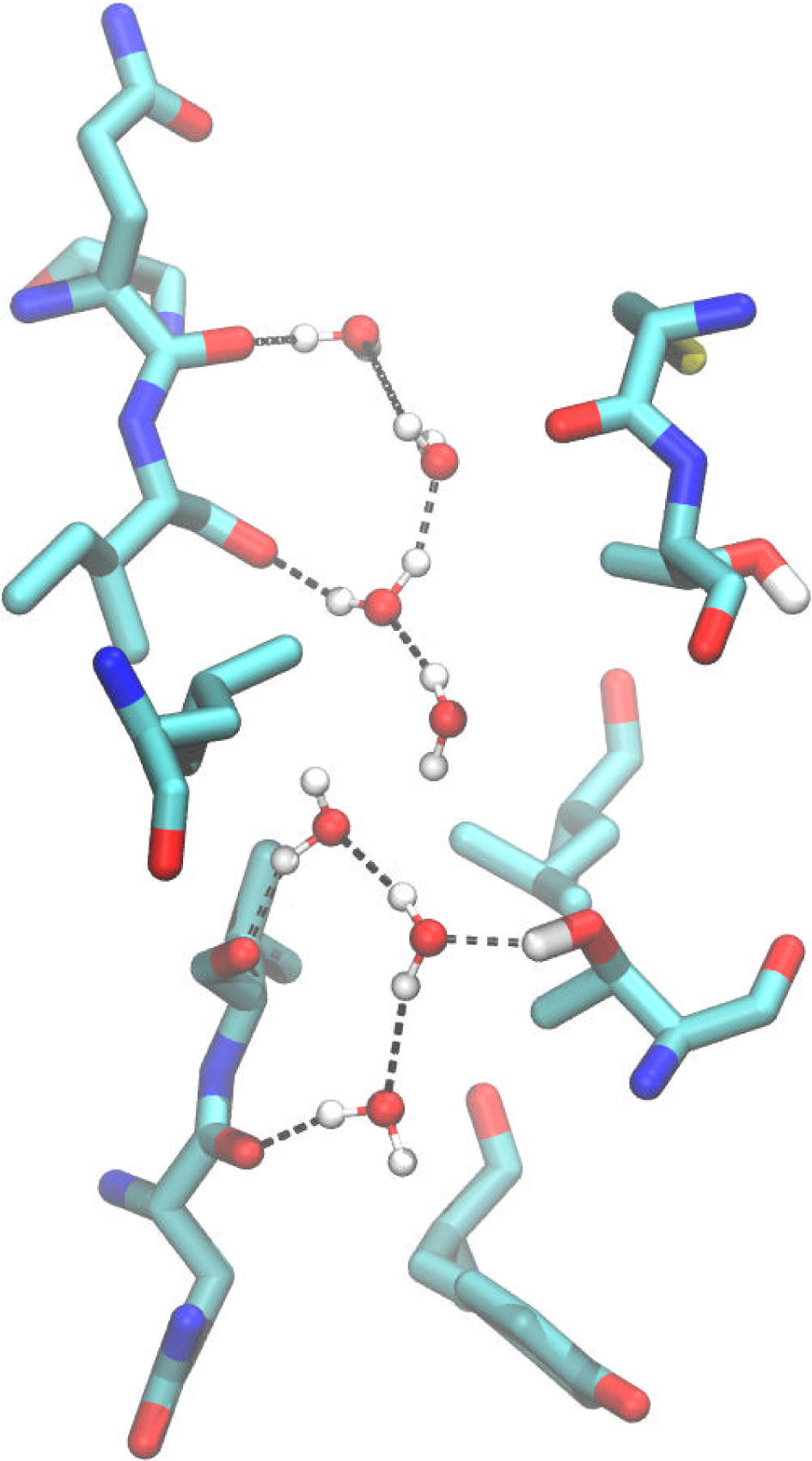

Supplement: Figure S4 — Hydrogen bonds between water molecules depicted in Figure 7b and pore residues. The figure is given separately for clarity. (TIF) [file pone.0082338.s004.tif]
