# Supplementary material for: Energetic and Molecular Water Permeation Mechanisms of the Human Red Blood Cell Urea Transporter B
Source: PLoS One. 2013 Dec 20;8(12):e82338. doi: 10.1371/journal.pone.0082338 (PMC3869693; doi:10.1371/journal.pone.0082338)
Supplement: File S1 — Construction and evaluation of structural model for the human sequence and simulation convergence. (PDF) [file pone.0082338.s005.pdf]

Supplemental materials to: Energetic and molecular water permeation mechanisms of the human red blood cell urea transporter B

Slim Azouzi[1-4], Marc Gueroult[1-4], Pierre Ripoche[1-4], Sandrine Genetet[1-4], Yves Colin Aronovicz[1-4], Caroline Le Van Kim[1-4], Catherine Etchebest[1-4], and Isabelle Mouro-Chanteloup[1-4].

[1]Institut National de la Transfusion Sanguine, Paris, France F-75739;

[2]Inserm, UMR_S665, Paris, France F-75739;

[3]Université Paris Diderot, Sorbonne Paris Cité, Paris, France;

[4]Laboratory of Excellence GR-Ex.

Construction and Evaluation of structural model for the human sequence

The homology model of the human sequence was based on the bovine structure (pdb: 4EZC), which was solved at 2.36 Å resolution. The sequence alignment was based on HMM profiles available in PFAM database. The resulting alignment showed that both sequences share more than 83% identity on the overall length, the identity being even larger in the pore region defined by Levin et al (92%). We also tested ProbCons that is considered as a very good tool for alignment [1]. The resulting alignment was identical. Thus, as discussed and shown in different papers the identity score is high enough to be confident in the quality of the model [2,3]. In addition, to evaluate the model quality, geometry analyses on model and X-ray structure were performed with Procheck software. The Ramachandran plot gave 95.1% of residues in the most favoured regions, 4.6% for additional allowed region and 0.3% in generously allowed regions for the model, compared to 94.6%, 5.1% and 0.3% for the X-ray structure, respectively. Consequently, the quality of the model is at least, as good as the X-ray structure. We also evaluated the quality of our model using ProQM [4] and compared to the score obtained for the X-ray structure. The scores were highly similar and large enough for considering the model as a good one (0.71 for the model and 0.72 for the X-ray structure) Figure S1.

Simulation Convergence:

Convergence of each simulation was characterized by root mean square deviation (RMSD) calculation (Figure S2) from to the structure at time t=0, i.e after minimization and equilibration with position restraints on the protein. Simulations on human model converged after 75ns of simulation and the two replica showed equivalent deviation for the human model. Regarding simulation of X-ray bovine structure, deviation was slightly smaller. The simulation convergence was also assessed using Principal Component analysis (PCA).

PCA analysis was first performed on each half of simulation to evaluate the convergence of each simulation for the human model. Cα atoms were examined in the analysis that was restricted to the equilibrium part of simulation (75-200ns). N-ter and C-ter of structure were discarded from the analysis due to the high flexibility of these regions (Figure 5), which would demand larger conformational sampling. Analysis of the contribution of each PC showed that the 10 first PCs characterize more than 90% of fluctuation (93% and 95% for MD1 and MD2, respectively).

Firstly, the overlap between the two halves of each simulation was calculated using the covariance overlap methodology developed by Hess [5] . Covariance overlaps are 0.70 and 0.69 within MD1 and MD2 respectively, which indicates that convergence was reached within each MD.

Secondly, when PCA was performed on the whole set of data, i.e. concatenating the two trajectories, but focusing on the transmembrane region, the overlap equals 0.35. Hence, the two replica explore different conformational spaces. This value is similar to the one observed by Grossfield et al. [6] for other membrane proteins simulated on similar timescale (100ns). When considering the transmembrane domain of a monomer, the overlap was increased significantly (0.47).

The inner products given in Figure S3 illustrate the similarity in the different cases.

Finally, when focusing on the residues defining the pore, the overlap was 0.60, showing the convergence of pore fluctuations, which is also exemplified by the similarity of permeation parameters.

In conclusion, the mechanism we examined, i.e. water transport seems to occur on a timescale compatible with the present simulation time and we were able to obtain reproducible results. It seems that large conformational changes are not required for water transport to occur.

1. Nuin PA, Wang Z, Tillier ER (2006) The accuracy of several multiple sequence alignment programs for proteins. BMC Bioinformatics 7: 471.
2. Forrest LR, Tang CL, Honig B (2006) On the accuracy of homology modeling and sequence alignment methods applied to membrane proteins. Biophys J 91: 508-517.
3. Wilson CA, Kreychman J, Gerstein M (2000) Assessing annotation transfer for genomics: quantifying the relations between protein sequence, structure and function through traditional and probabilistic scores. J Mol Biol 297: 233-249.
4. Ray A, Lindahl E, Wallner B (2010) Model quality assessment for membrane proteins. Bioinformatics 26: 3067-3074.
5. Hess B (2002) Convergence of sampling in protein simulations. Phys Rev E Stat Nonlin Soft Matter Phys 65: 031910.
6. Grossfield A, Feller SE, Pitman MC (2007) Convergence of molecular dynamics simulations of membrane proteins. Proteins 67: 31-40.
